# Increased Compulsivity in Adulthood after Early Adolescence Immune Activation: Preclinical Evidence

**DOI:** 10.3390/ijerph18094684

**Published:** 2021-04-28

**Authors:** Santiago Mora, Elena Martín-González, Ángeles Prados-Pardo, Pilar Flores, Margarita Moreno

**Affiliations:** Department of Psychology and Health Research Centre, University of Almería, Carretera de Sacramento s/n, 04120 Almería, Spain; emg771@ual.es (E.M.-G.); app717@ual.es (Á.P.-P.); pflores@ual.es (P.F.)

**Keywords:** inhibitory control, compulsivity, immune activation, behavior, preclinical models, early life adversity

## Abstract

Immune activation during early developmental stages has been proposed as a contributing factor in the pathogenesis of neuropsychiatric conditions such as obsessive-compulsive disorder, attention-deficit/hyperactivity disorder, and autism in both human and animal studies. However, its relationship with the vulnerability to inhibitory control deficit, which is a shared feature among those conditions, remains unclear. The present work studied whether postnatal immune activation during early adolescence, combined with exposure to early-life adverse events, could lead to adult vulnerability to impulsive and/or compulsive behaviors. Male Wistar rats were exposed to lipopolysaccharide (LPS) in early adolescence at postnatal day 26 (PND26). During peripuberal period, half of the animals were exposed to a mild stress protocol. In adulthood, behavioral assessment was performed with the aid of the sustained attentional 5-choice serial reaction time (5-CSRT) task, schedule-induced polydipsia (SIP), and open-field locomotor activity and novelty reactivity. Rats exposed to LPS showed more compulsive responses than their control counterparts on 5-CSRT task, although no differences were observed in SIP or locomotor responses. Our study contributes to the knowledge of the relationship between immune activation and inhibitory control deficit. Future studies should aim to disentangle how, and to what extent, immune activation impacts behavior, and to understand the role of early life mild stress.

## 1. Introduction

Inhibitory control is an executive function that mediates our behavior by attention and reasoning, thus enabling us to inhibit or control predominant responses under environmental demands [1]. Failures in this process result in inhibitory control deficit, whose two main manifestations are impulsivity and compulsivity [2,3]. Impulsivity involves performing actions or making decisions that might result in potentially negative consequences for the individual and lack an appropriate forethought [4]. On the other hand, and although significantly less studied than impulsivity [3,5], compulsivity is an interesting phenomenon found across several neuropsychiatric disorders, such as schizophrenia, autism, addiction, and attention-deficit/hyperactivity disorder (ADHD), and it is a core feature in obsessive-compulsive disorder (OCD) [6,7]. Compulsivity can be defined as a perseveration of a response that is irresistible and inappropriate to the individual and unavoidable despite its negative consequences [8]. The implication of dopamine, serotonin and fronto-striatal circuitry on inhibitory control deficit has been extensively studied [2,5,9]; however, the potential factors underlying the vulnerability towards this condition have been less extensively studied.

Human studies have revealed that one putative factor that has gained notoriety during the last decades is early immune activation [10,11,12]. Benros et al. [13] coined the term ‘Immune activation hypothesis’ to account for the increasing evidence that postulates that viral- or bacterial-induced immune activation during critical neurodevelopment stages is a crucial factor underlying vulnerability to neuropsychopathological disorders. Indeed, early immune activation (during infancy and puberal period) has been linked to OCD, tic disorders, and Tourette’s syndrome [14], as well as ADHD [15,16], anorexia nervosa [17,18], depression [19] and autism [20,21]. Interestingly, these conditions share common features concerning inhibitory control deficit [10,12], and incardinate within the impulsive-compulsive spectrum.

A strong interest in studying the phenomenon with the aid of preclinical animal models under laboratory conditions has been raised (for review, see: [22]). Among the several preclinical models, one of the most studied is exposure to lipopolysaccharide (LPS), a cell wall component of Gram-negative bacteria that stimulates the production and release of pro-inflammatory cytokines and is used to mimic the immune activation. In preclinical studies, LPS has been shown to induce long-term changes in behavior, immune biomarkers and brain plasticity. Either following prenatal or early infancy exposure, LPS strongly affects a broad range of cognitive and behavioral processes, including those that present comorbidity in impulsive-compulsive spectrum disorders: for example, a deficit in the attenuation of acoustic startle reflex assessed by prepulse inhibition (PPI) [23,24,25,26,27,28], a proposed biomarker of schizophrenia; increased anxiety and fear [25,28,29,30,31], and impaired social interaction [32,33,34]. Moreover, LPS impaired learning and memory [25,27,28,31,35]; and motor activity, showing a decreased [35,36,37,38] or increased [28] locomotor activity and worse motor coordination [36]. LPS exposure affected immune signaling by: a higher interleukin 1β, 2 and 6 expression (IL-1β, IL-2 and IL-6) [24,29,30,35] in plasma, increased paired IG-like receptor B (PirB) levels in hippocampus [39], enhancement of in vitro mitogen-stimulated lymphocytes proliferation [23], and elevated tumoral necrosis factor α (TNFα) levels in serum [30,35] have been reported. LPS also altered neuroendocrine markers, such as elevated corticosterone and decreased testosterone and luteinizing hormone (LH) levels in plasma [29,30,40]. Finally, the effects of LPS induced neuroplasticity changes, such as: higher brain-derived neurotrophic factor plasma levels (BDNF) [34], reduced synaptophysin expression in cortex [27,39] and hippocampus [39], poorer myelination in basolateral amygdala, hippocampus, and orbitofrontal cortex [28], astrocyte hypertrophy and microglial activation [24], increased glial fibrillary acidic protein (GFAP) expression in dentate gyrus and CA3 [39], and reduced dopaminergic levels in striatum [34] and nucleus accumbens [27] have been found. Thus, immune activation with LPS creates a vulnerability that, under certain conditions, can lead to neurobiological and behavioral alterations. However, although some studies have linked LPS to increased stereotyped behavior [32] and increased marble burying [41], to our knowledge, no studies have specifically addressed the relationship between immune activation with LPS and inhibitory control deficit with the aid of specific behavioral tasks as it has been done with Group-A Streptococcus (GAS) antigen [42].

Another important hypothesis to consider is that immune activation could create a latent vulnerability triggered by stress in the adult period [43], since stress during critical developmental stages is considered a crucial factor in vulnerability to neuropsychopathological disorders [44]. In fact, preclinical work in animal models has combined an early immune challenge with peripuberal stress in rodents [45], showed a synergistic effect of stress and immune activation on behavior, such as a decreased PPI and an increased amphetamine-induced locomotor activity compared to the other groups. Such intriguing possibility is especially relevant in inhibitory control deficit, where no apparent disturbances may exist. Thus, exploring the putative relationship between early immune activation and stress underpinning impulsive-compulsive urges further research. 

The present study aims to investigate whether early adolescence immune challenge using LPS in rats, in combination with exposure to mild stress during adolescence, can affect adult behavior and create a vulnerability to inhibitory control deficit. We hypothesized that challenged animals will differ from their control counterparts in terms of impulsive and/or compulsive behaviors, and that interaction between LPS exposure and stress might be found. The results are discussed in terms of neuro-immune regulation of behavior, how LPS seems to induce a long-term vulnerability, and the role that this latent vulnerability can play during adulthood.

## 2. Materials and Methods

### 2.1. Subjects 

48 male Wistar rats (Janvier, Le Genest-Saint-Isle, France) were used in this study. The animals arrived in the laboratory at postnatal day (PND) 21, right after weaning, and were housed four rats per cage (50 × 35 × 20 cm) at 22 °C, with a 12:12-h light–dark cycle (lights off at 07:00 h), with environmental enrichment (wooden blocks and PVC pipe tubes) and food and water provided ad libitum. Before the behavioral assessment, rats were gradually reduced to 85% of their free-feeding body weight through controlled feeding, and their body weights were maintained at this level of deprivation throughout the experiment. Food was provided by daily feedings of lab chow approximately 30 min after each experimental session. All the testing was performed between 09:00 and 14:00 h. All procedures were conducted according to the Spanish Royal Decree 53/2013 on the protection of experimental animals and the European Directive 2010/63/EU, and approved by the University of Almería Animal Research Committee. The authors declare that the research shows commitment to the Replacement, Reduction, Refinement (3Rs) principles.

### 2.2. Experimental Design

**Figure 1:**
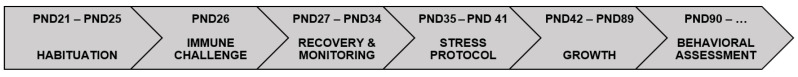
Experimental procedure illustrated in a timeline. Animals were assigned to four experimental conditions concerning exposure to lipopolysaccharide (LPS) and stress: saline-control (SAL/S-), LPS-control (LPS/S-), saline-stress (SAL/S+), or LPS-stress (LPS/S+), and habituated to the laboratory until PND26, where the immune challenge took place. The following days the functional observational battery (FOB) was employed to assess illness symptoms. Between postnatal days 35 and 41 (PND35 and PND41), four stressors were applied on non-consecutive days. After stress protocol, rats were left undisturbed until behavioral assessment started at PND90. Once finished, animals were sacrificed by decapitation and their brains removed and stored at −80 °C for further processing.

Each animal was assigned to one of four experimental conditions: saline-control (SAL/S-), LPS-control (LPS/S-), saline-stress (SAL/S+), or LPS-stress (LPS/S+), with a n of 12 in each group. Early adolescence LPS immune challenge was performed at PND26 (for an outstanding review of rat age and its relation to humans, see: [46]; for animal LPS model at early adolescence see: [47]), and exposition to stressors occurred on alternate days between PND35 and PND41 in both cohorts [45]. All animals were left undisturbed (but for handling, weighing, or assessment of signs of sickness), with food and water available ad libitum, between the exposition to stressors and once concluded until PND90, when behavioral assessment started. Figure 1 describes the experimental timeline; further details can be found in the following paragraphs.

#### 2.2.1. Lipopolysaccharide Immune Challenge

After 5 days of habituation to the laboratory conditions, at PND26, animals were administered with either lipopolysaccharide (LPS, from *Escherichia coli*, 0111: B4, Sigma-Aldrich, Madrid, Spain), solved in 0.9% pyrogen-free saline, at a dose of 0.1 mg/kg and a volume of 1 mL/kg (*n* = 24), or with saline alone (*n* = 24). LPS dose was selected according to previous evidence [28,34,35,41] and exposure time was based on Ariza-Traslaviña and cols. [47]. To assess any possible sign of severe sickness in the animals a short version of the Functional Observation Battery (FOB) [48,49], was used at 24, 48, and 72 h, 7 and 14 days after exposure to LPS. The assessed parameters were presence/absence of lacrimation, salivation, diarrhea, piloerection, tremor, and flat or hunched posture; and weight and temperature were recorded. 

#### 2.2.2. Exposure to Stress

Between PND35 and PND41, half of the animals (SAL/S+ and LPS/S+ groups) were exposed to four different mild stressors on four alternate days. The choice of stress agents was based on previous evidence [45]: 

Exposure to wet litter: 400 mL of tap water were added to the sawdust bedding of a new cage to which the rats were transferred and in which they remained for 10 h within the active (dark) phase of the light-dark cycle.

Exposure to repeated changing of home cages: rats were changed from the home cage to a new cage with fresh bedding, repeated for a total of five times on the dark phase on a non-predictable basis (irregular time intervals).

Exposure to forced swimming: for two sessions of one minute separated by a 3-min interval, animals were placed in a circular black tank (diameter: 29.5 cm, height: 62.5 cm) placed on a brightly lit testing room, filled with water (temperature: 21 °C, depth: 30 cm) and within the dark phase. Between sessions, the animals were kept in a climatized waiting box containing dry sawdust bedding, and after each swimming session, they were dried with paper towels. After the two sessions had concluded, they were dried again and returned to their home cages.

Exposure to restraint stress: animals were gently placed inside a flexible plastic cone with a hole on its narrow end to facilitate oxygen supply. The other end was closed with a cable tie surrounding the portion of the tail nearest to the body, with special care to not apply too much pressure which might damage the tail. They were left restrained inside cages with fresh bedding in a brightly lit room for 35 min, after which they were taken out, cleaned, handled for approximately 30 s, and returned to their home cages.

### 2.3. Behavioral Assessment

#### 2.3.1. 5-Choice Serial Reaction Time (5-CSRT) Task

Subjects were required to respond to light flashes randomly presented in one of five different spatial locations [50]. Subtle modifications from previous studies [1,51] were made for pre-training and training. A detailed description of the apparatus and procedure has been provided previously [52,53]. The animals underwent the 5-CSRT task until they met a specific performance criterion, consisting of <50 correct responses, <80% accuracy, and <20% omissions, with a stimulus duration (SD) of 1 s. Each daily session comprised 100 discrete trials, and performance stability was achieved after about 40 sessions. Each test session started with the illumination of the chamber house light and the food magazine light, where a food pellet was dispensed. Its collection started the first trial, and subsequently, the next trials were self-initiated by an entry into the food magazine; then, as the food magazine light was extinguished, a 5 s fixed inter-trial interval (ITI) started. After the ITI, a light stimulus of 1 s duration was randomly presented in one of the apertures at the rear wall of the operant chamber. Nose-poke responses in the illuminated aperture within 5 s, the limited hold (LH), were registered as correct responses and rewarded by the delivery of a food pellet in the magazine feeder, which was signaled by its illumination. Response errors were registered as either omissions (when a failure to respond within the LH occurred), errors of commission (when the response was made into a wrong location), or premature responses (when made during the ITI, before the visual stimulus was presented). Any of these response errors were followed by a 5-s time-out period of darkness, where no food was delivered. Additional responses in a given aperture between the correct response and the food collection was registered as a perseverative response, which was not punished by a time-out period; the food collection started the next trial. While an entry to the food magazine following the delivery of the food pellet or a timeout period started the next trial, an entry after a premature response resulted in restarting the same trial. Each session ended after either 100 trials or 30 min. In order to facilitate the task acquisition, the SD was progressively shortened from 8 s to 1 s: in stage 1, SD = 8 s, LH = 10 s and ITI = 5 s; the SDs of stages 2 to 5 were 6 s, 2.5 s, 1.25 s, and 1 s, respectively, with constant LH and ITI of 5 s. Animals went through stages if they were successful to achieve at least 50 correct trials, >80% accuracy, and <20% omissions. Measures recorded were: accuracy (correct trials/correct trials + incorrect trials × 100), number of correct responses, number of incorrect responses, % omission (number of omissions/correct responses + incorrect responses + omissions × 100), number of premature responses, number of perseverative responses, and latencies (in seconds) to (a) correct response (between stimulus presentation and correct nose poke), (b) incorrect response (between stimulus presentation and incorrect nose poke) and (c) reward (between correct response and collection of the food pellet in the magazine). Baseline measurements were calculated as the mean of three consecutive sessions where criterion was met (>80% accuracy, <20% omission). After acquisition and baseline, further impulsivity assessment was performed by challenging the animals with a long ITI (LITI) session, during which the waiting interval between trial initiation and light stimulus presentation was increased to 10 s. This procedure is used as a tool for assessing impulsivity since the increase in the waiting time exacerbates impulsive behavior as measured by premature responses. This is a sensitive and valid approach for exploring individual differences in impulsivity and has been previously used to dissociate high and low impulsive individuals [54,55]. Moreover, animals were tested under extinction condition (EXT), where pellet dispensers were disconnected and, thus, correct responses were not reinforced, to further explore compulsive behavior [53].

#### 2.3.2. Schedule-Induced Polydipsia (SIP) Procedure

Testing was performed in twelve operant SIP chambers (32 × 25 × 34 cm) (MED Associates, St. Albans, VT, USA). Detailed apparatus description has been previously provided [53]. A computer and commercial software Med PC (Cibertec SA, Madrid, Spain) were used to scheduling and recording of the experimental events. Prior to SIP, a baseline test of two successive days was used to measure water ingestion, with sixty food pellets (Noyes 45 mg dustless reward pellets; TSE Systems, Bad Homburg, Germany) were available together, and the amount of water consumed by each animal in 60 min was recorded. Then, after one session of habituation and adaptation to the SIP chambers, animals underwent a fixed time 60-s (FT-60s) food pellet presentation schedule in 60-min sessions. Water bottles, placed in the wall opposite the food magazine and containing 100 mL of freshwater, were available throughout the whole session. The measures recorded for each animal were (a) total water amount (in milliliters) removed from the bottle, (b) total number of licks, and (c) total number of entries to food magazine (for further details, see [56]).

#### 2.3.3. Spontaneous Locomotor Activity and Novelty Reactivity

Animals underwent an Open-field test in Plexiglas activity cages (39× 39×15 cm), where photocell beams were sent to the computer (Cibertec SA, Madrid, Spain) through an interfaced microcomputer VersaMax^®^ Animal Activity Monitoring System (AccuScan Instruments Inc., Columbus, OH, USA). For a previous description of the task, see: [53]. Locomotor activity was assessed based on the number of photocell beam breaks. Without previous habituation to the activity cages, the locomotor response of the rats to a novel environment was recorded in the activity cages. Locomotor behavior was quantified in twelve 5-min intervals over a 60-min period after placing animals in the test cage for both total (total activity) and interval by interval (habituation) activity.

### 2.4. Data Analysis

Data from the sickness assessment was analyzed using one-way repeated measures analysis of variance (ANOVA) with six/three measures (for weight: baseline, 24 h, 48 h, 72 h, 7 d, and 14 d; for temperature and piloerection: 24 h, 48 h, and 72 h after LPS exposure) and one inter-group factor (LPS); when appropriate, one-way analyses were performed to analyze effects within one measure. 5-CSRT task acquisition data were analyzed using two-way repeated-measures ANOVA with five measures (SD8, SD6, SD2.5, SD1.25, and SD1) and two between-subjects factors (LPS and stress). 5-CSRT task baseline data (general performance, speed measures, and inhibitory control measures) was analyzed using two-way ANOVA with two between-subjects factors (LPS and stress). 5-CSRT task variables manipulations were analyzed with two-way repeated-measures ANOVA with two measures (baseline and LITI/EXT) and two between-subjects factors (LPS and stress). SIP was analyzed using two-way repeated-measures ANOVA with twenty measures (session 1 to session 20) and two between-subjects factors (LPS and stress). Locomotor activity was analyzed using two-way repeated-measures ANOVA with twelve measures (sample 1 to sample 12) and two between-subjects factors (LPS and stress). When appropriate, post hoc analyses were performed using Bonferroni correction. The statistical significance was set at *p* < 0.05 (non-significant reported as *n.s.*), and the effect size was reported when appropriate; the partial eta-squared values were reported and considered as small (0.01), medium (0.06), or large (0.14) according to the recommendations in Cohen et al. [57]. All analyses were carried out using the Statistica^®^ software version 6.0 (Statsoft, Hamburg, Germany) and a computer (Cibertec SA, Madrid, Spain).

## 3. Results

**Figure 2:**
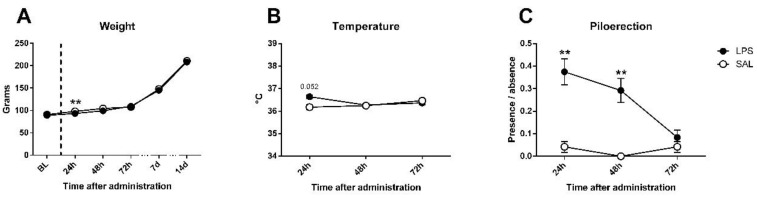
Sickness assessment with the aid of functional observational battery (FOB) in saline (SAL) and Lipopolysaccharide (LPS) treated animals. (**A**) Bodyweight evolution of experimental subjects: no differences existed in baseline, but LPS induced a deficit in weight gain in treated animals 24 h after administration; differences disappeared at 48 h and groups remained equal until the last assessment (14 days later). (**B**) Temperature data: a trend towards significance was present 24 h after administration, where LPS-treated group exhibited higher temperature, but not 48 or 72 h later. (**C**) Piloerection presence/absence (index from 0 to 1): a strong effect was found on LPS-treated animals 24 and 48 h after immune challenge, and disappeared 72 h later. Asterisk represents LPS effect. Data is represented as mean ± standard error of the mean (SEM).

For the sickness assessment, there was found an effect on weight gain (Figure 2A), expectable in growing animals (day effect: F5,230 = 2717.45; *p* < 0.0001; partial η^2^ = 1); however, although no differences existed in baseline weight (F1,46 = 0.393; n.s.), treated and untreated animals differed 24 h after LPS administration (F1,46 = 9.69; *p* < 0.01; partial η^2^ = 0.861), but differences disappeared at 48 h (F1,46 = 2.5; n.s.). Regarding the temperature data (Figure 2B), an interaction between day and LPS was observed (F2,92 = 3.267; *p* < 0.05; partial η^2^ = 0.608), not found in day (F2,92 = 1.201; n.s.) nor LPS effect (F1,46 = 0.855; n.s.) alone; an effect close to significance was found 24 h after injection in temperature (F1,46 = 3.972; *p* = 0.052; partial η^2^ = 0.496), but disappeared at 48 h (F1,46 = 0.019; n.s.). Piloerection data showed LPS effect (F1,46 = 17.116; *p* < 0.001; partial η^2^ = 0.981), but non-significant trends by day (F2,92 = 2.473; *p* = 0.089; partial η^2^ = 0.485) or interaction (F2.92 = 2.875; n.s.) existed. One-way comparisons showed that differences between groups in piloerection can be found at 24 (F1,46 = 9.316; *p* < 0.01; partial η^2^ = 0.848) and 48 h (F1,46 = 9.47; *p* < 0.01; partial η^2^ = 0.853), but disappeared at 72 h (F1,46 = 0.343; n.s.) after LPS injection (Figure 2C). Nevertheless, no evidence of the remaining signs of sickness (tremor, hunched/flat posture, lacrimation, salivation, or diarrhea) were found in any of the animals. Details can be found in Table 1.

No changes were found in body weight before and after stress exposure protocol, neither due to exposure to LPS (F1,44 = 0.752; n.s.) or stress (F1,44 = 0.372; n.s.), although an overall day session, expectable in growing animals, existed (F1,44 = 7884.4; *p* < 0.001; partial η^2^ = 0.994).

### 3.1. 5-CSRT Task

#### 3.1.1. Acquisition and Baseline Performance

**Figure 3:**
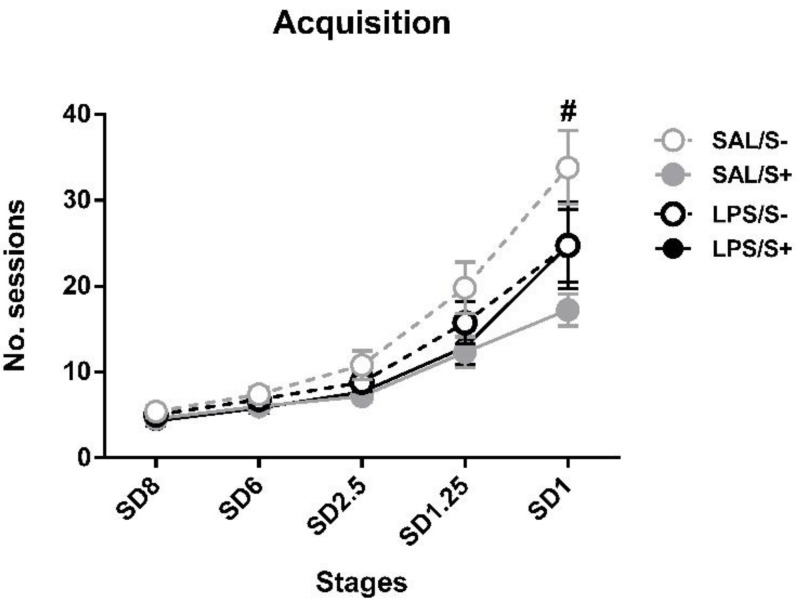
5-choice serial reaction time task (5-CSRT task) acquisition in saline-control (SAL/S-), LPS-control (LPS/S-), saline-stress (SAL/S+), or LPS-stress (LPS/S+) groups (*n* = 12). Mean number of sessions to criterion are represented at several stimulus durations (SD): 8, 6, 2.5, 1.25, and 1 s; at SD = 1, SAL/S+ (# symbol) group needed significantly fewer sessions than SAL/S- group to reach criterion. Data are represented as mean ± SEM.

Regarding the acquisition in 5-CSRT task (Figure 3), SAL/S- rats needed a mean of 33.83 ± 4.30 sessions to reach SD1 criteria, while LPS/S- needed 24.75 ± 5.06, SAL/S+ needed 17.25 ± 1.87 and LPS/S+ needed 24.75 ± 4.23. ANOVA revealed significant differences in acquisition, as stress (F1,44 = 5.633; *p* < 0.05; partial η^2^ = 0.641), stress × session interaction (F4,176 = 3.067; *p* < 0.05; partial η^2^ = 0.8) and session × LPS × stress (F4,176 = 3.569; *p* < 0.01; partial η^2^ = 0.863) effects were found. 

Post hoc analyses revealed that, while no differences existed at SD8, SD6 or SD2.5 and SD1.25, at SD1 the SAL/S+ group needed significantly less sessions to reach criterion than SAL/S- animals (*p* = 0.00006) Nevertheless, no general LPS (F1,44 = 0.283; n.s.), LPS × stress interaction (F1,44 = 2.622; n.s.) or LPS × session (F4,176 = 0.1; n.s.) effects were found.

**Figure 4:**
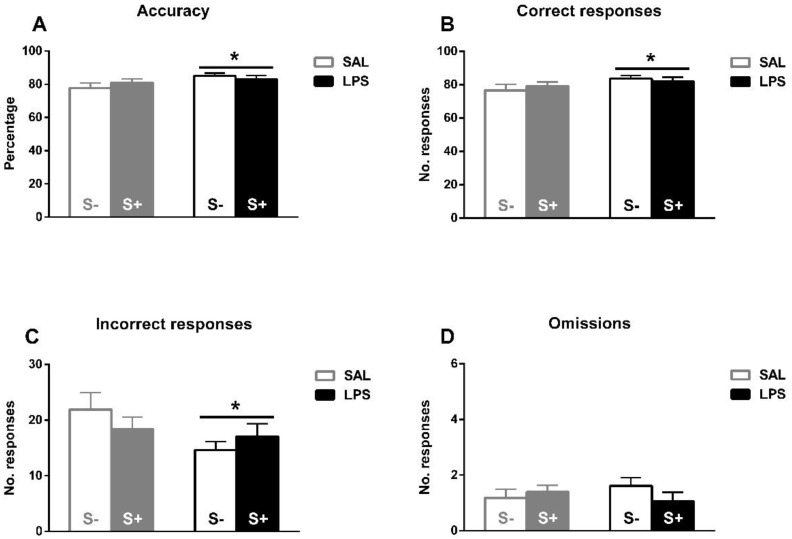
5-CSRT task baseline performance in saline-control (SAL/S-), LPS-control (LPS/S-), saline-stress (SAL/S+), or LPS-stress (LPS/S+) groups (*n* = 12). (**A**) Mean accuracy. (**B**) Mean correct responses. (**C**) Mean incorrect responses. (**D**) Baseline mean omissions. Asterisks represent LPS effect: exposed animals a better overall performance in terms of higher accuracy, more correct responses, and less incorrect responses. Data are represented as mean ± SEM.

Under baseline conditions (Figure 4A–D), the ANOVA revealed a better task performance induced by exposure to LPS. A significant increase in accuracy by LPS (F1,44 = 4.289; *p* < 0.05; partial η^2^ = 0.526), but not by stress (F1,44 = 0.056; n.s.) or their interaction (F1,44 = 1.569; n.s.) is found. In correct responses, a LPS effect was also found (F1,44 = 4.082; *p* < 0.05; partial η^2^ = 0.506), but no effect from stress (F1,44 = 0.031; n.s.) or their interaction (F1,44 = 0.672; n.s.). Similarly, concerning incorrect responses, a LPS effect was observed (F1,44 = 4.174; *p* < 0.05; partial η^2^ = 0.515), but no stress (F1,44 = 0.066; n.s.) or interaction (F1,44 = 1.939; n.s.) effects were found. No effect from LPS (F1,44 = 0.022; n.s.), stress (F1,44 = 0.352; n.s.), or interaction (F1,44 = 1.788; n.s.) were revealed in omissions. 

Table A1 shows the speed measures. In latency to correct responses, there was no effect from LPS (F1,44 = 0.34; n.s.), stress (F1,44 = 0.138; n.s.) or their interaction (F1,44 = 1.531; n.s.). Similarly, in latency to incorrect responses there was no effect from LPS (F1,44 = 1.006; n.s.), stress (F1,44 = 0.01; n.s.) or their interaction (F1,44 = 0; n.s.). Finally, no effects were found in latency to reward as well, nor from LPS (F1,44 = 0.432; n.s.), nor from stress (F1,44 = 1.033; n.s.) or from their interaction (F1,44 = 1.322; n.s.).

#### 3.1.2. Inhibitory Control Assessment

**Figure 5:**
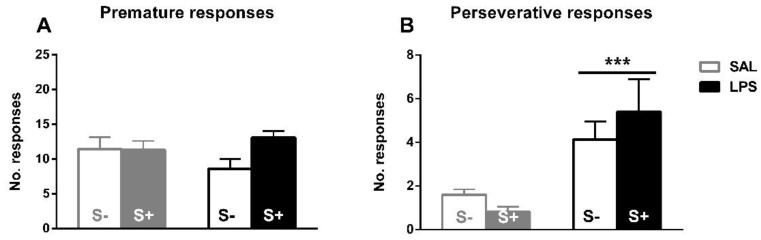
5-CSRT task inhibitory control variables in saline-control (SAL/S-), LPS-control (LPS/S-), Scheme 12. (**A**) Baseline mean premature errors. (**B**) Baseline mean perseverative errors: asterisks represent that animals exposed to LPS (LPS/S- and LPS/S+) had a significantly more perseverative behavior than controls (*p* < 0.001). Data is represented as mean ± SEM.

The assessment of inhibitory control, perseverative and premature responses in the 5-CSRT task (Figure 5). Animals exposed to LPS showed an increased compulsive behavior, as an effect exists in perseverative responses (F1,44 = 17.679; *p* < 0.001; partial η^2^ = 0.984); however, no effect of stress (F1,44 = 0.088; n.s.) or interaction (F1,44 = 1.469; n.s.) were found. On the contrary, there was no significant effect from LPS exposure (F1,44 = 0.163; n.s.), stress (F1,44 = 2.793; n.s.) or their interaction (F1,44 = 3.102; n.s.) in impulsivity measured by premature responses.

**Figure 6:**
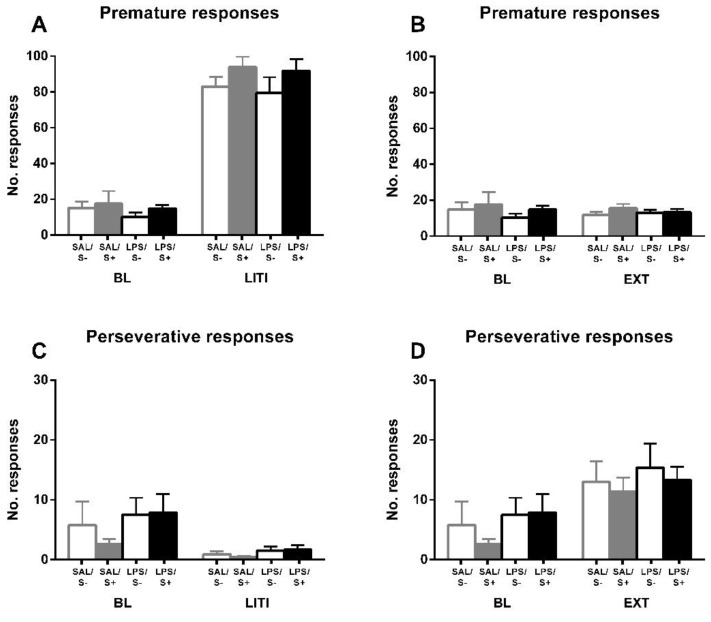
5-CSRT task variables manipulations in saline-control (SAL/S-), LPS-control (LPS/S-), saline-stress (SAL/S+), or LPS-stress (LPS/S+) groups (*n* = 12). Performance of animals is shown under long inter-trial interval (LITI) and extinction (EXT) conditions for premature (**A**,**B**) and perseverative (**C**,**D**) responses, compared to baseline (BL). As it was expected, the LITI condition significantly increases premature responses and decreases perseverative responses in all groups; extinction significantly increases perseverative responses in all groups without affecting premature responses. Data is represented as mean ± SEM.

For further exploring the individual differences in inhibitory control, and according to the approach of several authors [52,54,55] previously mentioned, performance under LITI and EXT conditions was measured (Figure 6). LITI dramatically increased impulsive behavior, assessed by premature responses (condition effect: F1,44 = 684.41; *p* < 0.0001; partial η^2^ = 1), in all groups equally (LPS effect: F1,44 = 0.861; n.s.; stress effect: F1,44 = 2.956; n.s.; interaction: F1,44 = 0.043, n.s.), while significantly decreasing perseverative responses (condition effect: F1,44 = 15.45; *p* < 0.001; partial η^2^ = 0.97) in all groups equally (LPS effect: F1,44 = 2.216; n.s.; stress effect: F1,44 = 0.299; n.s.; interaction: F1,44 = 0.52; n.s.), thus vanishing the baseline effect from LPS (F1,44 = 3.007; n.s.). EXT did not have any effect on premature responses (condition effect: F1,44 = 0.02; n.s.; LPS effect: F1,44 = 1.524; n.s.; stress effect: F1,44 = 1.273; n.s.; interaction: F1,44 = 0.02; n.s.); however, it significantly increased perseverative responses (condition effect: F1,44 = 33.638; *p* < 0.0001; partial η^2^ = 0.999) in all groups equally (LPS effect: F1,44 = 1.325; n.s.; stress effect: F1,44 = 0.436; n.s.; interaction: F1,44 = 0.109; n.s.), leading the baseline LPS effect to disappear as well (F1,44 = 0.541; n.s.).

### 3.2. Schedule-Induced Polydipsia

**Figure 7:**
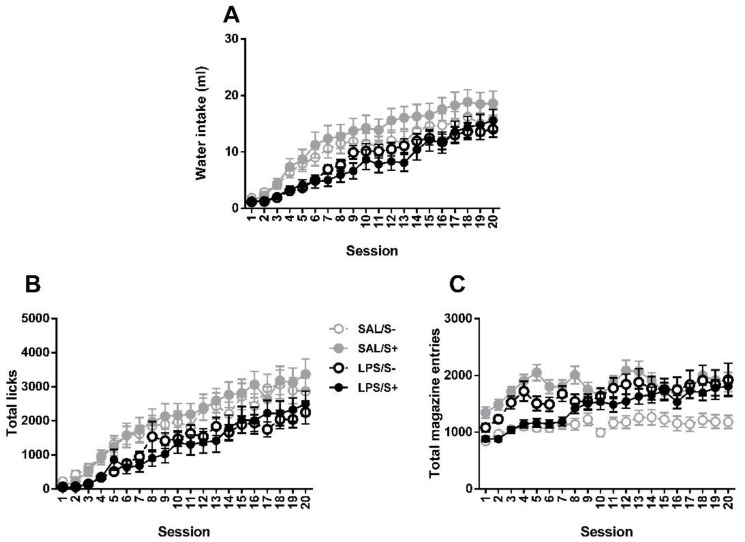
Schedule-induced polydipsia (SIP) acquisition in saline-control (SAL/S-), LPS-control (LPS/S-), saline-stress (SAL/S+), or LPS-stress (LPS/S+) groups (*n* = 12). (**A**) Mean water intake (in milliliters), (**B**) total licks, and (**C**) total magazine entries are shown across 20 sessions. No effect of LPS or stress was found in any of the measures. Data are represented as mean ± SEM.

Two-way repeated measures ANOVA revealed no significant differences between groups in SIP acquisition (Figure 7) by LPS exposure (F1,44 = 2.175; n.s.) or stress (current effect: F1,44 = 0.082: n.s.) in water intake (interaction: F1,44 = 0.308; n.s.), while an overall day effect was found (F19,836 = 43.18; *p* < 0.0001; partial η^2^ = 1). Same pattern is found in total licks, with no effect from LPS (F1,44 = 2.591; n.s.), stress (F1,44 = 0.01; n.s.) or their interaction (F1,44 = 0.029; n.s.); and a day effect was also found (F19,836 = 31.511; *p* < 0.0001; partial η^2^ = 1). In total magazine entries, day (F19,836 = 7.452; *p* < 0.0001; partial η^2^ = 1), LPS × stress (F1,44 = 5.031; *p* < 0.05; partial η^2^ = 0.592) and LPS × day (F19,836 = 1.785; *p* < 0.05; partial η^2^ = 0.97) effects were revealed, but no LPS (F1,44 = 0.11; n.s.) or stress (F1,44 = 1.207; n.s.) effects existed.

### 3.3. Spontaneous Locomotor Activity

**Figure 8:**
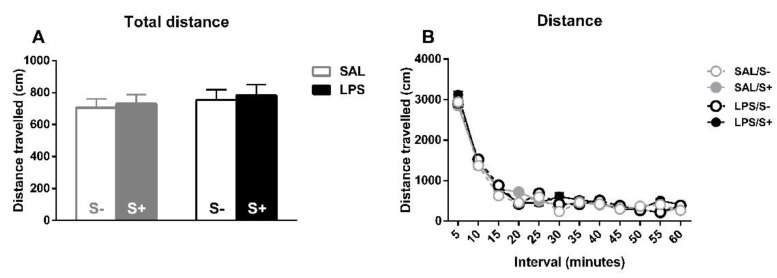
Locomotor activity in saline-control (SAL/S-), LPS-control (LPS/S-), saline-stress (SAL/S+), or LPS-stress (LPS/S+) groups (*n* = 12). (**A**) Total distance traveled by the animals on the whole session. (**B**) Reactivity to novel environment (distance traveled on each five-minute interval). No differences were found concerning LPS or stress in any of these measures, although overall habituation to the cage can be observed in terms of a decrease of distance traveled. Data are represented as mean ± SEM.

For spontaneous locomotor activity (Figure 8), the total distance travelled (Figure 8A) and the habituation to the novel environment (Figure 8B) were evaluated for free-moving animals. For total distance, no effect from LPS (F1,44 = 2.061; n.s.), stress (F1,44 = 1.081; n.s.) or their interaction (F1,44 = 1.046; n.s.) were found. Concerning reactivity to novelty, there was a significant overall decrease in movement as time passed (sample effect: F11,484 = 210.02; *p* < 0.0001; partial η^2^ = 1), but no effect from LPS (F1,44 = 1.042; n.s.), stress (F1,44 = 0.29; n.s.) or their interaction (F1,44 = 0.001; n.s.) was found.

## 4. Discussion

The present study investigated the effects of early adolescent immune activation with LPS and adolescent mild stress on inhibitory control during adulthood. Animals exposed to LPS showed an increased compulsive behavior compared to their control counterparts, in terms of significantly more perseverative responses in the 5-CSRT task, even though an overall better baseline performance existed. This effect disappeared under LITI, where all groups showed decreased perseverative responses, and under extinction, where all groups showed increased perseverative responses. No differences were found on the remaining 5-CSRT task measures, nor in SIP or locomotor activity. Our data on adult animals exposed to LPS at PND26 (which correspond with the early adolescent period in relation to humans, see: [46]), points toward a possible implication of early adolescent immune activation on adult inhibitory control deficit.

The functional observational battery (FOB) showed some short-term signs of sickness after LPS exposure. Although the dose and exposure protocol for inducing immune activation were chosen based on previous evidence [28,32,33,34,35,47], applying the FOB for assessing its effectiveness is useful for two purposes: 1) reassuring that, on our experiment, the immune challenge effectively took place, and 2) as a tool for monitoring sickness behavior, enabling us to check subjects closely so as to avoid any risk and, if necessary, intervene to guarantee the least necessary suffering on the experimental subjects. There was a significant decrease in weight gain 24 h after challenge, which disappeared at 48 h. Food intake and appetite are crucial indexes of health [58], and, at this early developmental stage, rats increase their weight in a very rapid manner [59], thus any impact on body weight shall be taken as a relevant sign of sickness. Similarly, the temperature is commonly raised when immune activation takes place, due to infectious or inflammatory challenges, even with agents such as LPS, which does not represent actual risk to the organism [60,61,62,63,64,65]. A non-significant temperature raise was found in LPS exposed animals when compared to controls; it disappeared after 48 h. Last, piloerection is seen as one relevant sign of concern in laboratory rats [48], related, at the same time, with fever and temperature loss [66,67]; this was the measure where the strongest effect was seen, with differences between challenged and non-challenged animals 24 and 48 h after. It is noteworthy that this effect was present up to 48 h later, when even the weight differences had disappeared. These effects of sickness behavior are in line with the previous evidence demonstrating the systemic immune impact of LPS, such as increased IL-1β, IL-2, IL-6, and TNFα levels [24,29,30,35].

Exposure to LPS created a long-term vulnerability to compulsivity, as shown by the 5-CSRT task. Compared to their saline-exposed counterparts, animals exposed to LPS showed significantly more perseverative responses, which are registered when animals continue to respond at the apertures even when food presentation is signaled and are an index of compulsive-like behavior [51,52]. This dramatic behavioral effect is an expression of top-down cognitive control failure [53,68], and in line with previous studies that have reported effects on related behaviors after early immune activation in rodents pre- or postnatally: exposure to LPS resulted in an increase in stereotypic behavior [32,33,41] and marble burying [32]. Interestingly, immune activation with LPS has been reported to induce alterations in PPI, and a PPI deficit has been consistently found [23,24,25,26,27,28]. This is crucial, since this deteriorated PPI is comorbid not only in schizophrenia, but also in other disorders linked to inhibitory control deficit, such as OCD, Tourette’s, depression, and substance abuse [69], and is found in preclinical models of compulsive behavior [70]. Other preclinical models of immune activation have reported similar behavioral effects: exposure to GAS antigen has been shown to increase impulsive responses in 5-CSRT task [42] and a reversal spatial hole-board task [71], as well as stereotyped behaviors [72,73,74] and increased marble burying [72,73]. Moreover, Holloway et al. [75] reported an increased serotoninergic 5-HT2A/C receptor agonist DOI-induced head-twitch responses after Poly I:C exposure.

Interestingly, one of the most cardinal features across the Pediatric Autoimmune Neuropsychiatric Disorders Associated with Streptococcal infection (PANDAS), which shares early immune activation as a crucial factor with all these preclinical models, is a poor inhibitory control: it is well documented that GAS infections during infancy and adolescence multiply by 2 the risk of suffering OCD, Tourette´s and tic disorders, while recurrent episodes multiply by 3 [14]. Moreover, this infection has been linked to other impulsive-compulsive spectrum disorders, such as ADHD [15,16], anorexia nervosa [17,18], depression [19] and autism [20,21]; for review, see: [10,12]. There were no differences, however, in premature responses, which is another manifestation of poor inhibitory control [51] Contrary to compulsive perseverative responses, premature responses are an index of impulsivity, since they reflect a maladaptive loss of the inhibitory control of highly prepotent responses [51,52]. Although both phenomena are expressions of inhibitory control deficit, they are independent and, thus, can or not manifest together [2].

Once all the baseline measures were acquired, we manipulated inter-trial interval (ITI) and reward delivery, which are relevant modifications of the task for a more detailed assessment of premature and perseverative responses. Perseverative responses were significantly decreased under the long ITI (LITI) condition and increased under extinction in all groups, leading to the disappearance of the baseline effect of LPS exposure. On the contrary, premature responses were increased under LITI in all groups, while they were not altered by extinction. This data, although expectable, is crucial since it shows that subjects are sensitive to changes in the task configuration and reinforcement delivery, thus rejecting any possible explanation of the differences in baseline perseverative responses in those terms. These results, together with the 5-CSRT task acquisition and baseline performance data, strengthens the idea that inhibitory control, and not learning and/or memory deficits, underlie the differences in perseverative responses induced by early immune challenge.

Although animals exposed to LPS exhibited compulsive behavior in 5-CSRT task, no differences existed on schedule-induced polydipsia (SIP), neither in water milliliters nor in total likes. SIP is a well-stablished model of compulsivity, it meets the criteria for considering high drinker animals as compulsive subjects [76] and there exists a strong relationship between aberrant drinking in this paradigm and poor inhibitory control assessed on several paradigms [52,53,77,78,79,80,81], thus differences in water intake due to immune challenge would have been expected. However, as we discussed previously, compulsivity is a complex phenomenon, and different experimental models seem to assess different components or manifestations of the phenomenon. Thus, as perseverative responses on 5-CSRT task seem to reflect, as stated before, attentional bias/disengagement, the compulsive drinking behavior observed on SIP is more likely to reflect habit learning [82], where a repetitive performance without apparent adaptative function appears as a consequence of both a poor ability to inhibit action and a lack of sensitivity to goals [2].

No differences were found concerning total distance moved during locomotor activity. Existing literature has reported altered motor patterns after early immune challenge with LPS in rodents, concerning both decreased [35,36,37,38] and increased [28] locomotor activity, and worse motor coordination [36]. Since, although a differential motoric pattern would have been expected in the present study between animals exposed to LPS and their non-challenged counterparts, it is important to consider that these studies were performed with prenatal or very early exposure, while our exposure took place later, hence possibly reflecting different altered mechanisms. Other preclinical models of immune activation show a decrease in total distance covered [72,73,83] and impaired motor coordination [71,84], food manipulation [72,73], and narrow beam walking [72]. Moreover, in studies with human PANDAS patients, there is evidence of motor hyperactivity non-attributable to choreic movements [12,85,86] and deterioration in fine motor skills such as handwriting [86].

We found no differences in novelty reactivity between animals exposed to LPS and controls, nor concerning stress. Some authors have reported that animals selected as high reactive to novel environment are more impulsive [87], and spontaneously hypertensive rats (SHR), which are a genetic animal model of ADHD with a high impulsive phenotype, show locomotor hyperactivity in the open-field during habituation phase [88,89], which might imply a common trait linking impulsivity and locomotor hyperreactivity to novelty. Our finding, thus, along with our data in premature responses on 5-CSRT task, is consistent with a putative differential effect on impulsivity and compulsivity of exposure to LPS.

The present study leaves two surprising results: first, the apparently better performance in 5-CSRT task in animals exposed to LPS; second, the lack of a deleterious effect of stress. Under 5-CSRT task baseline conditions, animals exposed to LPS had a significantly better performance, in terms of higher accuracy, more correct responses and less incorrect responses, than saline-exposed animals, irrespectively from stress. Counterintuitive as it may seem, and despite wide evidence shows a poorer ability to learn new tasks such as Y- and Morris Water Maze [31,35] and object recognition [25] following immune challenge with LPS, and the attentional deficits in PANDAS [10,12,86], there exist reports of immune activation leading to improved learning and memory, such as increased performance on a spatial reversal learning task in mice transferred with streptococcus-induced antibodies [71], similarly to our result. Interestingly, the fact that the animals that perform better are the same that exhibit compulsive behavior can be explained in terms of different compulsivity expressions: when the major concept contributing to compulsivity is attentional bias (also known as disengagement), which refers to the ability to disengage and shift attention away from disorder-relevant stimuli, animals tend to create a rigid performance [2]. Thus, this ‘expression’ or component of compulsivity might have been shown in the present study by animals exposed to LPS, with an inflexible behavior that results in a higher accuracy at the cost of an increased rate of compulsive responses.

Concerning stress, an apparently beneficial effect was found in 5-CSRT task acquisition, where, although no differences existed in the earlier stages (from SD8 to SD1.25), at SD1 SAL/S+ group reached criterion significantly sooner than SAL/S- animals. This result is surprising given the aforementioned deleterious effect of stress in combination with immune activation reported by Giovanoli et al. [45]. Since learning rates of the SAL/S- control group are the expectable in a normative group (for instance, see: [52]), in the present study, mild stress might have had a facilitator role in the learning of the task. One putative explanation for the effect lies on the notion that, as some authors have argued, duration and intensity of adverse events might be crucial factors concerning task performance, thus stress can either benefit or worsen cognitive functions and, thus, adaptative behavior [90]. Given that our stress protocol, shared with the approach by [45], was intended to model a situation of sub-chronic mild stress, the first explanation might be plausible, and the animals exposed to stressful conditions could have shown an enhanced acquisition of the 5-CSRT task.

The mechanism underlying long-term changes in inhibitory control following immune activation, however, remains unclear. The evidence of an inflammatory response following exposure to LPS, in terms of elevated IL-1β, IL-2 and IL-6, and TNFα [24,29,30,35] is in line with previous studies in our lab that have found that early immune activation following exposure to GAS antigen resulted in long-term increased TNFα in nucleus accumbens and decreased IL-6 and IL-18 in prefrontal cortex, cytokines that were related to impulsive behavior in 5-CSRT task and delay-discounting task [42] and have been linked to GAS in both preclinical [91], and human [92,93] studies. Thus, it has been hypothesized that the CNS is impacted by an autoimmune response that initiates a cascade of effects that result in a loss of inhibition. Interestingly, dopamine, which is strongly implicated in top-down inhibitory control [68], seems to be especially affected by such inflammatory response after the increased blood-brain barrier permeability [94] and the activation of calmodulin (CaM) kinase II, which alters dopaminergic release (for review, see: [95]). Further studies should aim to disentangle the mechanism(s) through which immune activation results in deleterious behavioral effects.

## 5. Conclusions

In conclusion, the present study found that early adolescent immune challenge with LPS created a vulnerability to compulsive behavior, as assessed by the 5-CSRT task perseverative responses during adulthood. It also increased task accuracy on baseline compared to controls, and irrespectively of stress. These differences disappeared under LITI and extinction, where all groups showed a similar behavioral pattern concerning compulsivity and impulsivity, as assessed by premature responses. Nevertheless, no differences emerged on SIP, another paradigm used to assess inhibitory control, or in distance traveled in open-field locomotor activity. Last, no differences were found in novelty reactivity, which seems to be in line with the no differences observed in 5-CSRT task impulsivity. These data points to the idea that early adolescent immune activation might create a vulnerability which, under certain conditions, leads individuals to the development of inhibitory control deficit, such as, in this case, compulsive behavior. Also, it gives us an insight into how immune challenge seems to trigger an intricate set of biological alterations with complex behavioral outputs. More research is needed to elucidate how the immune response impacts a developing organism and how the result of this interaction is reflected when the individual faces environmental demands and adverse events.

## Figures and Tables

**Table 1:** Mean values of sickness assessment measures (weight, temperature and piloerection) 24, 48, and 72 hours (h), 7, and 14 days (s) after LPS exposure. No evidence of the remaining signs of sickness (tremor, hunched/flat posture, lacrimation, salivation, or diarrhea) was found. Data are represented as mean ± SEM.

	SAL/S-	SAL/S+	LPS/S-	LPS/S+
**Weight** 24 h	98.0 ± 0.87	98.16 ± 0.8	93.5 ± 1.04	92.91 ± 0.87
48 h	105.0 ± 0.7	103.75 ± 0.74	96.25 ± 3.47	102.16 ± 0.73
72 h	104.75 ± 4.88	111.0 ± 0.89	108.58 ± 1.0	110.33 ± 0.89
7 d	148.08 ± 1.12	147.75 ± 1.22	145.08 ± 1.17	145.0 ± 1.09
14 d	211.5 ± 1.43	210.08 ± 1.53	209.08 ± 1.9	207.83 ± 1.61
**Temperature** 24 h	35.65 ± 0.13	36.7 ± 0.11	36.48 ± 0.1	36.8 ± 0.11
48 h	35.92 ± 0.08	36.56 ± 0.09	36.54 ± 0.08	36.0 ± 0.08
72 h	36.45 ± 0.07	36.46 ± 0.09	36.72 ± 0.08	6.0 ± 0.07
**Piloerection** 24 h	0.08 ± 0.04	0.0 ± 0.0	0.16 ± 0.06	0.58 ± 0.08
48 h	0.0 ± 0.0	0.0 ± 0.0	0.25 ± 0.07	0.33 ± 0.08
72 h	0.0 ± 0.0	0.08 ± 0.04	0.08 ± 0.04	0.08 ± 0.04

## Data Availability

The data presented in this study are available on request from the corresponding author. The data are not publicly available due to privacy concerns.

## Appendix A

Table A1 Mean speed measures (latencies to correct responses, incorrect responses, and to reward in Scheme 12. No effect of LPS, stress, or interaction was found in any of the measures. Data are represented as mean ± SEM.

**Table A1:** 5-CSRT task speed measures.

	SAL/S-	SAL/S	LPS/S-	LPS/S+
**Latency to Correct**	54.1 ± 4.7	57.1 ± 3.8	56.4 ± 2.0	50.8 ± 2.9
**Latency to Incorrect**	25.0 ± 5.4	24.5 ± 3.1	20.8 ± 4.4	20.4 ± 4.6
**Latency to Reward**	72.4 ± 3.6	71.8 ± 4.9	70.2 ± 3.8	79.7 ± 6.1
